# Supplementary material for: Mitochondrial proteins: Potential pathophysiological mechanisms of malignant progression in HCC
Source: PLoS One. 2025 Aug 21;20(8):e0329209. doi: 10.1371/journal.pone.0329209 (PMC12370077; doi:10.1371/journal.pone.0329209)
Supplement: Attachment — Submitted filename: Response to Reviewers.docx [file pone.0329209.s002.docx]

Dear Editors and Reviewers:

On behalf of my co-authors, we thank you very much for giving us an opportunity to revise our manuscript. We appreciate you very much for your valuable comments on our manuscript entitled “Mitochondrial proteins: potential pathophysiological mechanisms of malignant progression in HCC”. Your comments are very helpful in improving our paper, and they are significant guidance to our researchers. We have studied the suggestions carefully and have provided point-by-point responses in the letter below. We hope our responses adequately all editorial concerns and those of the reviewers, and that our manuscript is now acceptable for publication in your journal.

Journal requirements:

1. Please ensure that your manuscript meets PLOS ONE's style requirements, including those for file naming. The PLOS ONE style templates can be found at

https://journals.plos.org/plosone/s/file?id=wjVg/PLOSOne_formatting_sample_main_body.pdf and <https://journals.plos.org/plosone/s/file?id=ba62/PLOSOne_formatting_sample_title_authors_affiliations.pdf>

Response: Thank you very much for your suggestions and considerations on the article. According to the requirements of your magazine (https://journals.plos.org/plosone/s/file?id=wjVg/PLOSOne_formatting_sample_main_body.pdf and https://journals.plos.org/plosone/s/file?id=ba62/PLOSOne_formatting_sample_title_authors_affiliations.pdf), we adjusted the format of the article (P1， P6-14).

2. Please provide additional details regarding participant consent. In the ethics statement in the Methods and online submission information, please ensure that you have specified what type you obtained (for instance, written or verbal, and if verbal, how it was documented and witnessed). If your study included minors, state whether you obtained consent from parents or guardians. If the need for consent was waived by the ethics committee, please include this information.

Response: Thank you very much for your suggestions and considerations on the article. We added a statement in the article that all participants obtained written informed consent (P18).

3. When completing the data availability statement of the submission form, you indicated that you will make your data available on acceptance. We strongly recommend all authors decide on a data sharing plan before acceptance, as the process can be lengthy and hold up publication timelines. Please note that, though access restrictions are acceptable now, your entire data will need to be made freely accessible if your manuscript is accepted for publication. This policy applies to all data except where public deposition would breach compliance with the protocol approved by your research ethics board. If you are unable to adhere to our open data policy, please kindly revise your statement to explain your reasoning and we will seek the editor's input on an exemption. Please be assured that, once you have provided your new statement, the assessment of your exemption will not hold up the peer review process.

Response: Thank you very much for your suggestions and considerations on the article. All the data used in the article are from free public databases. We added this point in the article (P18). Furthermore, we have clearly stated the website addresses of all databases in the article to facilitate readers' access to the data.

4. PLOS requires an ORCID iD for the corresponding author in Editorial Manager on papers submitted after December 6th, 2016. Please ensure that you have an ORCID iD and that it is validated in Editorial Manager. To do this, go to ‘Update my Information’ (in the upper left-hand corner of the main menu), and click on the Fetch/Validate link next to the ORCID field. This will take you to the ORCID site and allow you to create a new iD or authenticate a pre-existing iD in Editorial Manager.

Response: Thank you very much for your suggestions and considerations on the article. We added the ORCID iD of the corresponding author in the author information.

5. Please include a separate caption for each figure in your manuscript.

Response: Thank you very much for your suggestions and considerations on the article. All of our pictures have a separate caption in Figure legend（P22-23）.

6. Please review your reference list to ensure that it is complete and correct. If you have cited papers that have been retracted, please include the rationale for doing so in the manuscript text, or remove these references and replace them with relevant current references. Any changes to the reference list should be mentioned in the rebuttal letter that accompanies your revised manuscript. If you need to cite a retracted article, indicate the article’s retracted status in the References list and also include a citation and full reference for the retraction notice.

Response: Thank you very much for your suggestions and considerations on the article. After reconfirmation, we found that all the references were complete.

**Review Comments to the Author**

Reviewer #1: Modest sample size but well-conducted study that highlights the prognostic significance of MYO19, DNA2, and FDPS in HCC using human samplese and cellular lines No bibliographic evidence available on the subject.

Response: We are very grateful for your professional advice.Our understanding of the roles of MYO19, DNA2 and FDPS in the prognosis of HCC was mainly obtained through databases such as KM-plot. We are very sorry. Due to the relatively small sample size we verified (34 pairs), we are temporarily unable to use our samples for survival analysis. We have been continuously collecting specimens and plan to conduct further in-depth research.

Reviewer #2: Liu et al have documented the expression of mitochondrial proteins in HCC, out of which three proteins were significantly associated with prognosis. The manuscript is well written. There is no mention about the histological subtypes (Macrotrabecular massive, Steatohepatitic, Fibrolamellar, etc) of HCC included in the study. Was the histology reviewed 34 patients?

Response: First of all, thank you very much for your recognition and suggestions on this article. I consulted the histological classification of the pathology department of our hospital. Among the 34 pairs of HCC tissues, 25 cases were Macrotrabecular massive, 5 cases were adenoid, and 4 cases were Steatohepatitic. No special histological classification was found. We previously only focused on the pathological classification and never paid attention to the histological classification. We will strengthen the research on the histological classification in the future.

Some minor changes: Figure 6. J,K,L are survival analysis instead of correlation analysis.

Response: Thank you very much for the comments. We are very sorry for such a mistake in the article. We have corrected the incorrect representation in the captions (P23).
